# The influence of a previous implant-based breast reconstruction on postoperative sensation of the deep inferior epigastric artery perforator flap

**DOI:** 10.1007/s12282-024-01558-6

**Published:** 2024-04-06

**Authors:** Joep A. F. van Rooij, Ennie Bijkerk, René R. J. W. van der Hulst, Stefania M. H. Tuinder

**Affiliations:** 1 Department of Plastic, Reconstructive, and Hand Surgery, Maastricht University Medical Center (MUMC+), P. Debyelaan 25, 6229 HX Maastricht, Limburg The Netherlands; 2 Department of Plastic, Reconstructive, and Hand Surgery, Maastricht University Medical Centre (MUMC+), P.O. Box 5800, 6202 AZ Maastricht, The Netherlands

**Keywords:** Breast reconstruction, Breast sensation, Implant-based breast reconstruction, Breast implants, DIEP flap

## Abstract

**Background:**

Implants and DIEP flaps have different outcomes regarding postoperative breast sensation. When compared to the preoperative healthy breast, implant-based breast reconstruction (IBBR) negatively influences postoperative breast sensation. However, it is currently unknown whether a prior IBBR also influences postoperative sensation of a replacing DIEP flap. The goal of this cohort study is to evaluate the influence of an IBBR on the postoperative sensation of a replacing DIEP flap.

**Methods:**

Women were included if they received a DIEP flap reconstruction after mastectomy, with or without prior tissue expander (TE) and/or definitive breast implant. Sensation was measured at four intervals in 9 areas of the breast with Semmes–Weinstein monofilaments: T0 (preoperative, implant/no reconstruction), T1 (2–7 months postoperative, DIEP), T2 (± 12 months postoperative, DIEP), Tmax (maximum follow-up, DIEP). Linear mixed-effects models were used to investigate the relationship between an implant/TE prior to the DIEP flap and recovery of breast sensation.

**Results:**

142 women comprising 206 breasts were included. 48 (23.3%) breasts did, and 158 (76.7%) breasts did not have a TE/IBBR prior to their DIEP. No statistically significant or clinically relevant relationships were found between a prior implant/TE and recovery of DIEP flap breast sensation for the flap skin, native skin, or total breast skin at T1, T2, or Tmax. There were also no relationships found after adjustment for the confounders radiation therapy, BMI, diabetes, age, flap weight, follow-up, and nerve coaptation.

**Conclusions:**

An implant/TE prior to a DIEP flap does not influence the recovery of postoperative breast sensation of the DIEP flap.

## Introduction

The number of breast cancer patients is increasing annually [1]. Simultaneously, the percentage of women choosing breast reconstruction after mastectomy is increasing and has doubled from 13% in 1998 to 26% in 2007 and continues to rise [2, 3]. In 2017, 30–40% of women chose a reconstruction after mastectomy in the Netherlands [4, 5].

Two of the most common reconstructive options are implant-based breast reconstruction (IBBR) or autologous reconstruction, often by the deep inferior epigastric perforator (DIEP) flap [6–8]. Cosmetic outcomes have always played and still play an important role in the reconstructive result. However, functional outcomes, such as breast sensation, are gaining importance and are known to be paramount in improving quality of life after reconstruction [9, 10]. Previous research has shown that breast sensation after mastectomy and IBBR is impaired [11, 12]. In contrast, when accompanied by nerve coaptation, autologous reconstruction with a DIEP flap is shown to be superior in recovery of postoperative breast sensation [13–16]. van Rooij et al. recently showed that it takes approximately 1.5 years after switching an IBBR for a DIEP flap reconstruction for sensation to recover back to the levels of IBBR [17]. In spite of this, the influence of a previous breast implant on the sensation of the replacing DIEP flap remains unknown.

When considering a post-mastectomy reconstruction, the patient’s opinion plays the dominant role [18, 19]. Providing sufficient information on breast reconstruction is essential for patients in the process of shared decision-making. Since breast sensation plays a major role in improving quality of life, the goal of this study is to compare sensation of the breast after DIEP flap reconstruction in patients with and patients without prior IBBR. This enables evaluation of whether or not implants have an influence on the postoperative sensation of the replacing DIEP flap.

## Materials and methods

In this cohort study, we included data of all patients who received a DIEP flap reconstruction after their mastectomy, with or without a prior tissue expander (TE) and/or definitive breast implant between August 2016 and August 2018 in the Maastricht University Medical Center (MUMC+), Maastricht, the Netherlands. Patients with primary, secondary, and tertiary breast reconstruction were included. This study was approved by the local medical ethical review board. All participants have provided written informed consent. All procedures performed in studies involving human participants were in accordance with the ethical standards of the institutional and/or national research committee and with the 1964 Helsinki declaration and its later amendments or comparable ethical standards.

### Data collection/outcomes

From August 2016 up until August 2018, all patients who underwent a DIEP flap breast reconstruction and Semmes–Weinstein monofilament (SWM) sensation measurements at the Department of Plastic, Reconstructive and Hand Surgery in the MUMC+, Maastricht, the Netherlands, were registered in a database [20]. The database contains surgical information and individual procedural details, patients’ medical characteristics, timing of reconstruction, tumor pathology, preliminary oncological treatment and postoperative complications or reoperations of the flap, as well as results of pre and postoperative SWM sensation measurements.

For this study, we included all patients who had a DIEP flap breast reconstruction with or without prior IBBR and had data of pre and postoperative SWM measurements. Patients who received postoperative radiation therapy on their DIEP flap were excluded due to the effect on skin sensation. Patients who had both missing pre and postoperative breast sensation measurements were also excluded.

The 20-piece SWM kit was used with index values ranging from 1.65 to 6.65 (monofilaments with increasing diameter). Several colors represent different grades of skin sensation, from best to worst sensation: green, normal touch (1.65–2.83); blue, diminished light touch (3.22–3.61); purple, diminished protective sensation (3.84–4.31); red, loss of protective sensation (4.56–6.45); and red, deep pressure sensation only (6.65). Measurements were performed with the patient in supine position while having their eyes closed, and by either one of two researchers with extensive experience in sensation measurements with the SWM.

For the primary outcome, we compared the difference in breast sensation measured by use of the SWM between two groups: one group of patients with a DIEP flap breast reconstruction without prior IBBR and one group of patients with a DIEP flap breast reconstruction with prior IBBR. Women received preoperative sensation measurements 1 day prior to their reconstructive surgery (T0; either healthy breast [primary reconstruction patients], post-mastectomy skin [secondary reconstruction patients], or TE/IBBR [tertiary reconstruction patients]). The postoperative measurements were performed during three outpatient check-ups: a postoperative measurement moment 1 (T1; DIEP flap) including measurements between 2 and 7 months postoperatively; a postoperative measurement moment 2 (T2; DIEP flap) including measurements around 12 months postoperatively; and a postoperative measurement moment at the last outpatient check-up with maximum follow-up (Tmax; DIEP flap). Nerve coaptation was performed by coapting the sensory donor nerve on the DIEP flap, originating from the 10th to 12th intercostal nerve, to the anterior cutaneous branch of the third intercostal nerve at the recipient site.

**Fig. 1:**
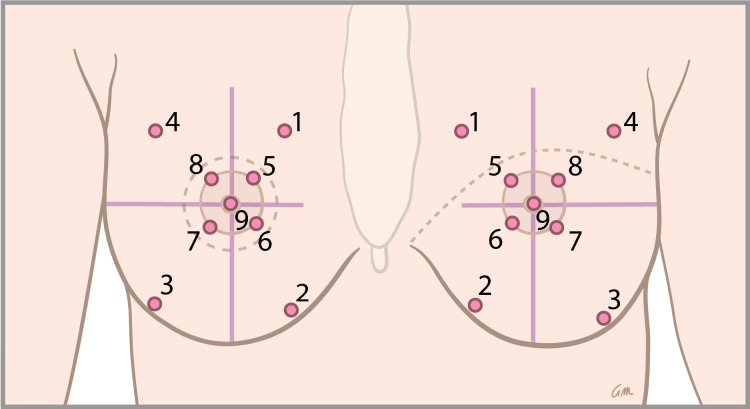
The breast divided in 9 areas for Semmes–Weinstein monofilament measurements (1–4 = peripheral breast, 5–9 = NAC) with scars after reconstruction as dashed lines following skin-sparing mastectomy (right) and following non-skin-sparing mastectomy (left)

**Fig. 2:**
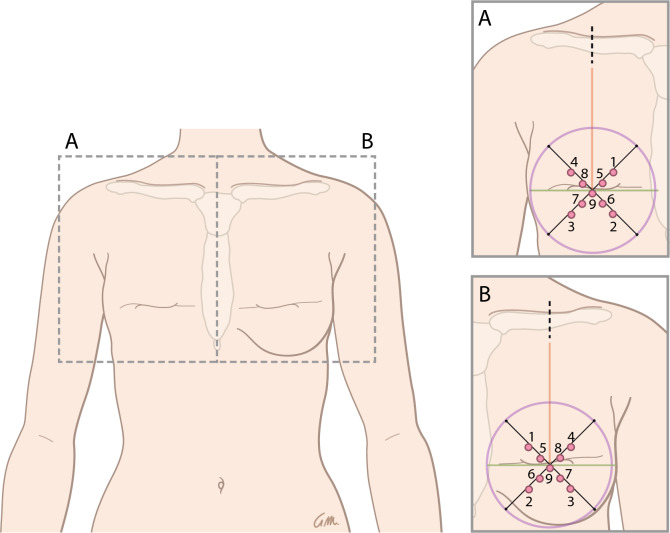
The breast without nipple immediately post-mastectomy (**A**) and after implant-based breast reconstruction (**B**) divided into 9 areas

For the sensation measurements, the breast was divided in 9 areas to be measured based on anatomical references (Fig. 1). In a healthy breast or a breast with an IBBR, measuring points 1–4 correspond to the breast skin, while points 5–9 correspond to the nipple-areolar complex (NAC). In the autologously reconstructed breast, depending on the type of mastectomy, measuring points 5–9 are generally considered flap skin if the patient had a history of skin-sparing mastectomy. For reconstruction after a non-skin-sparing mastectomy reconstruction, generally all measuring points except 1 and 4 are considered flap skin. Individual patient differences in flap and native skin were registered in case of intraoperative variability in skin quality and mastectomy flaps. The breast of patients without pre-existent or remaining nipple was divided into four quadrants with one vertical line from midclavicular to the caudal portion of the breast and one horizontal line perpendicular to the first. The lines cross each other at the level of the contralateral nipple and the intersection formed the center of the circle for measuring points 5–9 (Fig. 2).

### Statistical analysis

IBM SPSS Statistics 28 was used for statistical analysis (IBM Corp. Released 2021. IBM SPSS Statistics for Windows, Version 28.0. Armonk, NY: IBM Corp). Descriptive statistics were used for patients’ baseline characteristics. A mean and standard error for the SWM measurements was calculated for the four measurement moments T0, T1, T2, and Tmax. For each follow-up moment, means and differences with standard error were calculated for the two groups (DIEP flap with and DIEP flap without prior IBBR) for flap skin, native skin, and the total breast according to the corresponding measuring points. SWM values were visualized in scatterplots.

With over 5% of incomplete postoperative SWM measurements due to missing data, multiple imputation was used to prevent a loss of statistical power and decrease the likelihood of biased results. The number of imputations was set to 20 and predictive mean matching was used to draw the values to be imputed.

The logarithmically transformed values of the SWM were used for analysis, because they form normal distributed data, unlike their values in grams. Unit of analysis was the breast. Linear mixed-effects models were used to investigate relationships between an implant or TE prior to the DIEP flap reconstruction and recovery of breast sensation. Hence, the patient population consists of women with and without an IBBR prior to their DIEP flap. We corrected for the following confounders: radiation therapy, BMI, diabetes, age at operation, flap weight, follow-up, and nerve coaptation. To compare SWM values between the DIEP group with and without a prior implant, an independent samples t-test was used.

We performed an exploratory analysis to compare the postoperative breast sensation for reconstructed breasts with and without a nerve coaptation using the independent-samples t-test. These analyses were performed separately for the group with a prior IBBR and the group without a prior IBBR.

## Results

For this study we included 142 women, comprising 206 breasts in total. All women had a DIEP flap reconstruction. 158 of 206 breasts (76.7%) did not have an IBBR prior to the DIEP flap reconstruction. 48 of 206 breasts (23.3%) did have an IBBR prior to the DIEP flap (Table 1, baseline characteristics). The mean age of the women with a prior implant was 50.2 years. The mean age of women without a prior implant was 51.8 years. In the non-implant group, 110 patients (69.9%) had a primary autologous breast reconstruction and 48 patients (30.4%) had a secondary autologous breast reconstruction. Logically, all of the patients in the implant group had a tertiary autologous breast reconstruction.

**Table 1:** Baseline characteristics

Characteristic	Reconstruction without prior implant (%) (*n* = 158)	Missing	Reconstruction with prior implant (%) (*n* = 48)	Missing
Age (years)	50.2	–	51.8	–
Follow-up (months)				
T1 (mean)	4.6 (± 1.5; range 2–7)	15	4.6 (± 1.8; range 2–7)	6
T2 (mean)	12.3 (± 2.9; range 8–20)	22	14.1 (± 4.7; range 9–29)	6
Tmax (mean)	18.2 (± 5.7; range 9–34)	30	16.3 (± 5.5; range 9–29)	6
Reconstruction timing				
Primary	110 (69.6)	–	0 (0)	–
Secondary	48 (30.4)	–	0 (0)	–
Tertiary	0 (0)	–	100 (100)	–
Implants in place (months)	N.A	–	81.2 (± 73.0; range 4–327)	
Nerve anastomosis				
Yes	87 (55.1)	–	19 (39.6)	–
No	71 (44.9)	–	29 (60.4)	–
BMI (kg/m^2^)	27.5 (± 4.3)	3	26.7 (± 3.7)	–
Smoking		–		–
Yes	4 (2.5)		4 (8.7)	
No	154 (97.5)		44 (91.7)	
Diabetes		–		–
Yes	2 (1.3)		0 (0.0)	
No	156 (98.7)		48 (100.0)	
Radiation therapy		3		–
Yes	38 (24.5)		11 (22.9)	
No	117 (75.5)		37 (77.1)	
Chemotherapy		3		–
Yes	77 (49.7)		23 (47.9)	
No	78 (50.3)		25 (52.1)	
Hormone therapy		3		–
Yes	42 (27.1)		16 (33.3)	
No	113 (72.9)		32 (66.7)	
Necrosis mastectomy skin	12 (12.8)	64	0 (0.0)	19
*Breast complications*		2		1
Yes	19 (12.2)		7 (14.9)	
Venous congestion	2 (1.3)	1	1 (2.1)	1
Infection	14 (8.9)	1	4 (8.5)	1
Wound problems	32 (20.4)	1	5 (10.6)	1
Fat necrosis	9 (5.7)	1	2 (4.3)	1
Hematoma	9 (5.7)	1	3 (6.4)	1
Seroma	3 (1.9)	1	2 (4.3)	2
No	137 (87.8)		40 (85.1)	

Mean follow-up for the implant group was 4.6 ± 1.8 months for T1, 14.1 ± 4.7 months for T2, and 16.3 ± 5.5 months for Tmax. Mean follow-up for the non-implant group was 4.6 ± 1.5 months for T1, 12.3 ± 2.9 months for T2, and 18.2 ± 5.7 for Tmax.

For T1 (follow-up 2–7 months), T2 (follow-up around 12 months), and Tmax (maximum follow-up) we calculated crude and adjusted values for flap skin, native skin, and total breast skin. The p-value for all coefficients was >0.05 and thus no relationship was found between a previous implant and postoperative sensation of the replacing DIEP flap (Table 2).

**Table 2:** Linear mixed-effects models analysis of postoperative breast sensation and a previous implant

Time point	Number of breasts	Breast skin	Coefficient	95% CI (upper bound to lower bound)	*p* value
T1	206 (158 + 48)	Total			
		Crude	0.030	−0.20 to 0.26	0.80
		Adjusted^a^	−0.048	−0.31 to 0.21	0.72
	206 (158 + 48)	Flap			
		Crude	0.030	−0.20 to 0.26	0.80
		Adjusted^a^	−0.048	−0.31 to 0.21	0.72
	206 (158 + 48)	Native			
		Crude	0.027	−0.34 to 0.40	0.89
		Adjusted^a^	−0.020	−0.42 to 0.38	0.92
T2	206 (158 + 48)	Total			
		Crude	0.061	−0.19 to 0.31	0.64
		Adjusted^b^	−0.0051	−0.28 to 0.27	0.97
	203 (155 + 48)	Flap^c^			
		Crude	0.08	−0.22 to 0.38	0.60
		Adjusted^b^	−0.01	−0.32 to 0.30	0.95
	206 (158 + 48)	Native			
		Crude	0.12	−0.21 to 0.45	0.47
		Adjusted^b^	0.14	−0.24 to 0.52	0.48
Tmax	206 (158 + 48)	Total			
		Crude	0.088	−0.15 to 0.32	0.47
		Adjusted^b^	0.068	−0.27 to 0.41	0.69
	203 (155 + 48)	Flap^c^			
		Crude	0.10	−0.16 to 0.36	0.43
		Adjusted^b^	0.13	−0.25 to 0.51	0.50
	206 (158 + 48)	Native			
		Crude	0.2	−0.12 to 0.53	0.22
		Adjusted^b^	0.12	−0.32 to 0.55	0.60

*CI* confidence interval

^a^ Adjusted for radiation therapy, BMI, diabetes, age at operation, flap weight, and follow-up

^b^ Adjusted for radiation therapy, BMI, diabetes, age at operation, flap weight, follow-up, and nerve anastomosis

^c^ Two patients/three breasts missing postoperative flap values due to skin island removed in secondary procedure

**Fig. 3:**
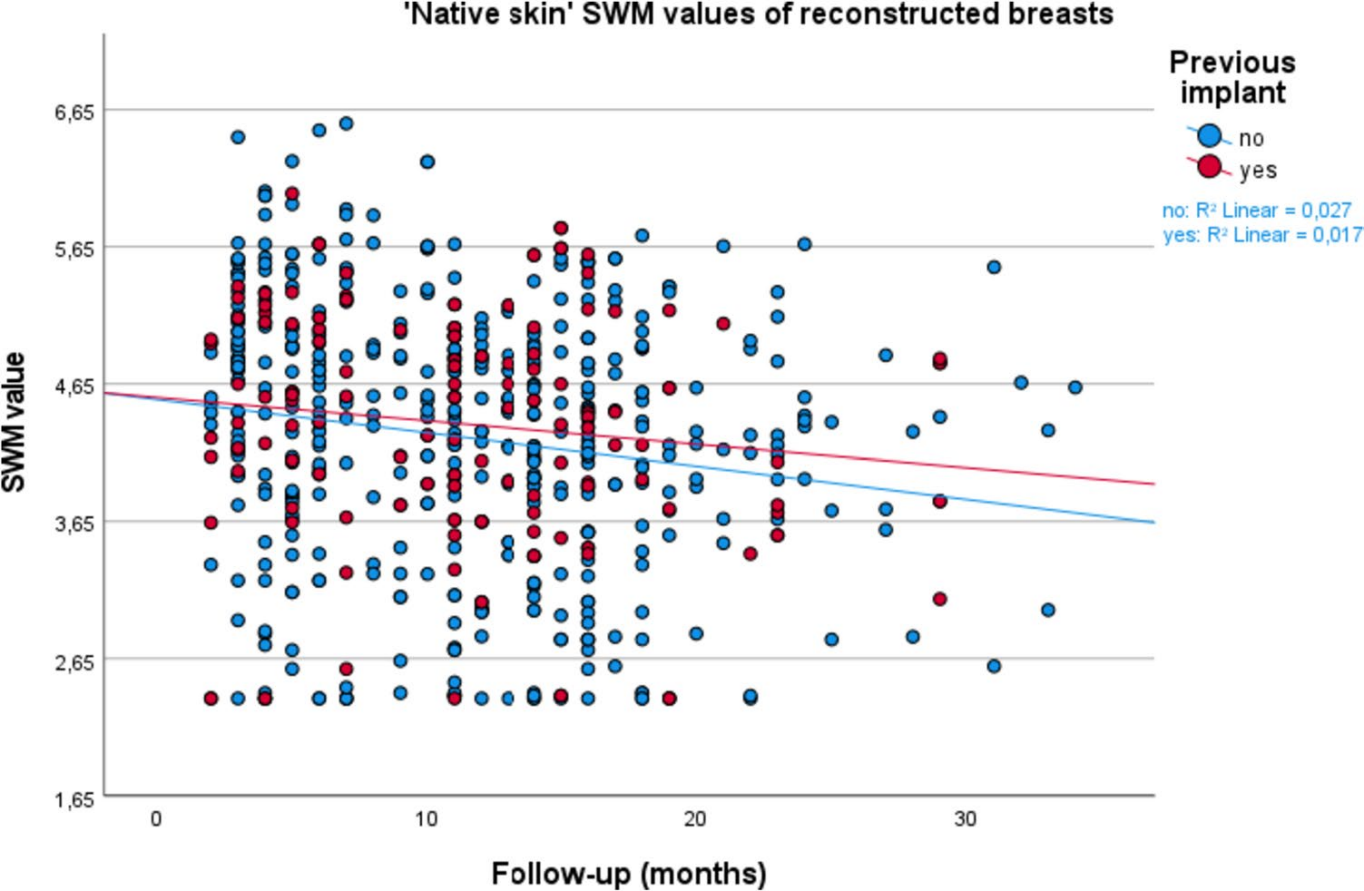
Scatterplot for ‘native skin’ Semmes–Weinstein monofilament (SWM) values, comparing patients with and without an implant prior to their DIEP flap breast reconstruction

**Fig. 4:**
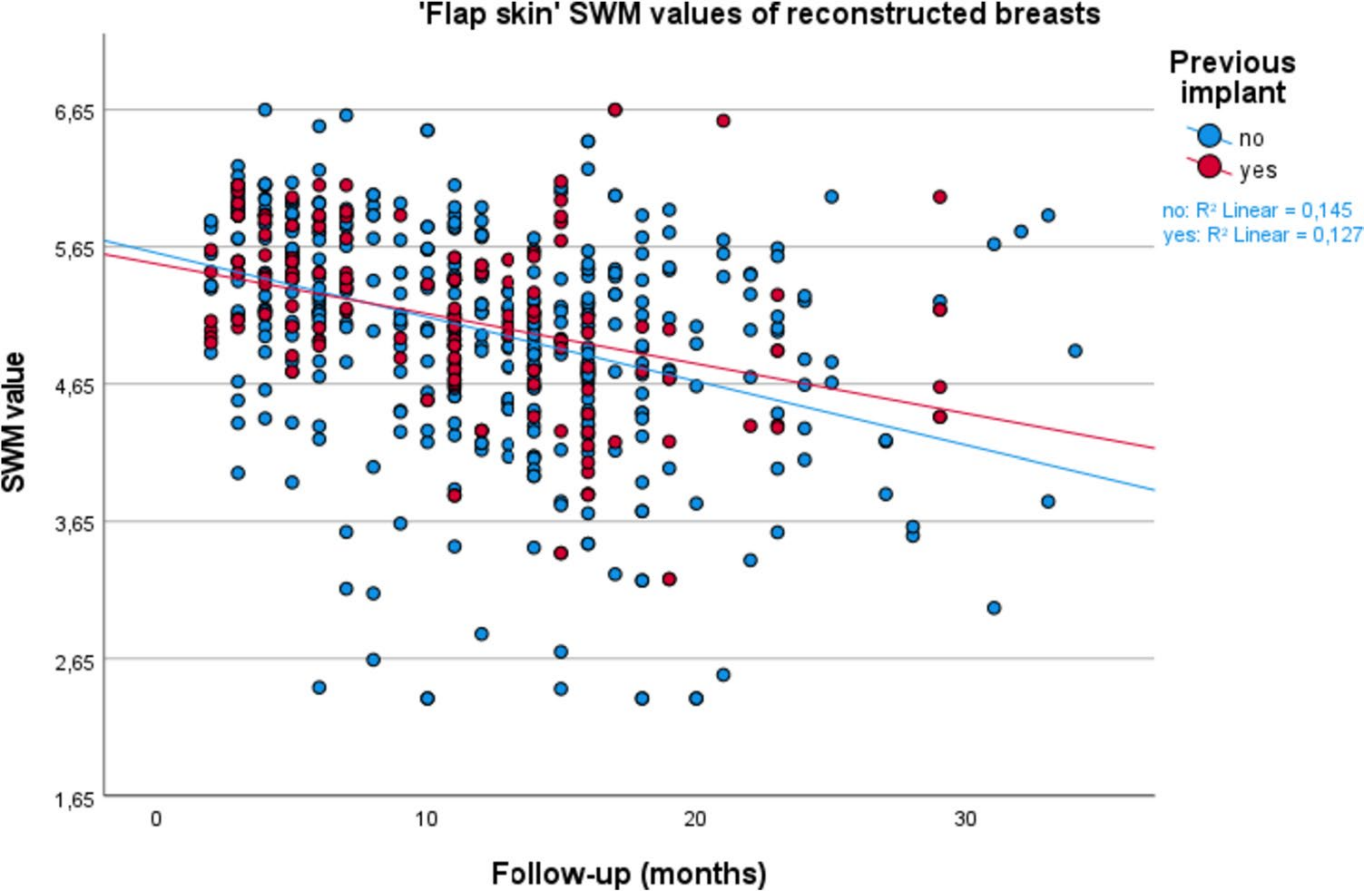
Scatterplot for ‘flap skin’ Semmes–Weinstein monofilament (SWM) values, comparing patients with and without an implant prior to their DIEP flap breast reconstruction

**Fig. 5:**
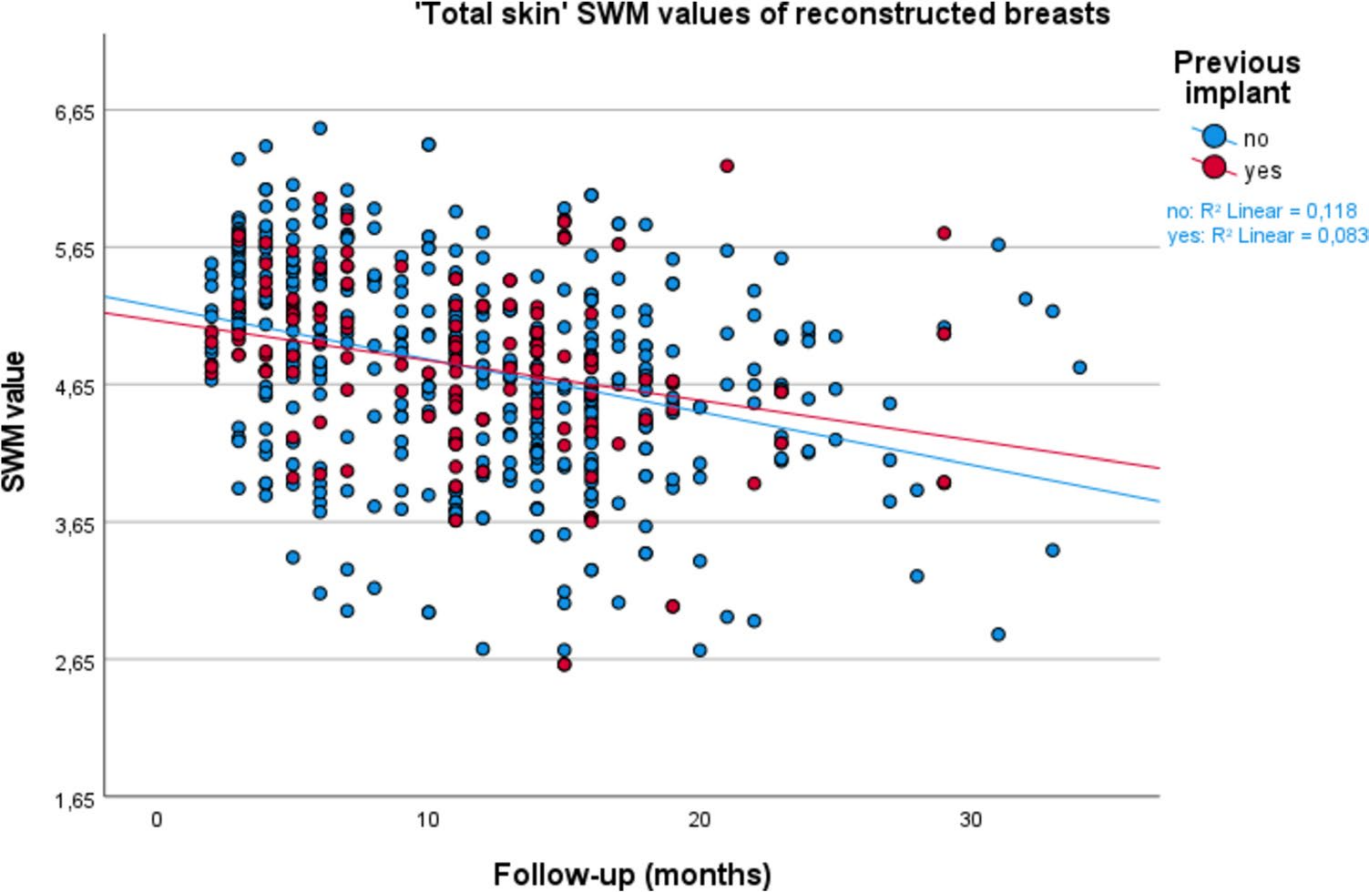
Scatterplot for ‘total skin’ Semmes–Weinstein monofilament (SWM) values, comparing patients with and without an implant prior to their DIEP flap breast reconstruction

Mean SWM value comparisons between the DIEP group with and the DIEP group without a prior implant are displayed in Table 3. At T0, prior to the DIEP, SWM values were significantly higher for the group with an implant compared to the group without an implant. This was the case for the native skin, flap skin, and total skin. At T1, T2, and Tmax, there were no statistically significant sensation differences left between the implant and non-implant group for the native, flap, and total skin. Scatter plots for the native skin, flap skin, and total skin have been plotted for the groups with and without an implant prior to their DIEP flap breast reconstruction and can be seen in Figs. 3, 4, and 5.

**Table 3:** SWM values and comparisons between the groups with and without an implant prior to their DIEP flap

	With implant	SWM category	Without implant	SWM category	Absolute difference (=with − without implant)	*p* value
T0 (*n* = without/with)						
Mean native (*n* = 158/48)	3.34		2.70		+0.64 (SE 0.11)	<0.001*
Mean flap^a^ (*n* = 158/48)	3.82		3.02		+0.80 (SE 0.14)	<0.001*
Mean total (*n* = 158/48)	3.69		2.90		+0.79 (SE 0.13)	<0.001*
T1 (*n* = without/with)						
Mean native (*n* = 158/48)	4.51		4.52		−0.01 (SE 0.18)	0.95
Mean flap (*n* = 158/48)	5.45		5.46		−0.01 (SE 0.11)	0.97
Mean total (*n* = 158/48)	5.06		5.11		−0.05 (SE 0.11)	0.64
T2 (*n* = without/with)						
Mean native (*n* = 158/48)	4.23		4.14		+0.09 (SE 0.16)	0.60
Mean flap^b^ (*n* = 155/48)	4.94		4.92		+0.02 (SE 0.14)	0.89
Mean total (*n* = 158/48)	4.64		4.65		−0.01 (SE 0.12)	0.93
Tmax (*n* = without/with)						
Mean native (*n* = 158/48)	4.29		4.09		+0.20 (SE 0.16)	0.22
Mean flap^b^ (*n* = 155/48)	4.88		4.77		+0.11 (SE 0.13)	0.43
Mean total (*n* = 158/48)	4.60		4.52		+0.08 (SE 0.12)	0.47

SE = standard error; SWM = Semmes–Weinstein monofilaments; SWM categories = green, normal touch (1.65–2.83); blue, diminished light touch (3.22–3.61); purple, diminished protective sensation (3.84–4.31); red, loss of protective sensation (4.56–6.45); and red, deep pressure sensation only (6.65)

^*^ *p* < 0.05 indicating statistical significance

^a^ Mean of preoperative breast areas corresponding to postoperative flap skin areas

^b^ Two patients/three breasts missing postoperative flap values due to skin island removed in secondary procedure

Mean SWM value comparisons between the DIEP flap breast reconstructions with and without nerve coaptation are displayed in Table 4 for the group with a previous implant and in Table 5 for the group without a previous implant.

**Table 4:** Breast sensation of DIEP flap reconstructed breasts with and without nerve coaptation for the group with a previous implant

	Without nerve coaptation	SWM category	With nerve coaptation	SWM category	Absolute difference (=without − with nerve coaptation)	*p* value
T1 (*n* = without/with)						
Mean native (*n* = 29/19)	4.69		4.23		−0.46 (SE 0.29)	0.11
Mean flap (*n* = 29/19)	5.45		5.46		+0.01 (SE 0.15)	0.95
Mean total (*n* = 29/19)	5.07		5.06		−0.01 (SE 0.16)	0.97
T2 (*n* = without/with)						
Mean native (*n* = 29/19)	4.25		4.19		+0.05 (SE 0.26)	0.84
Mean flap (*n* = 29/19)	5.05		4.78		+0.27 (SE 0.21)	0.18
Mean total (*n* = 29/19)	4.68		4.58		+0.09 (SE 0.20)	0.65
Tmax (*n* = without/with)						
Mean native (*n* = 29/19)	4.27		4.31		+0.04 (SE 0.27)	0.89
Mean flap (*n* = 29/19)	4.92		4.81		−0.11 (SE 0.23)	0.62
Mean total (*n* = 29/19)	4.59		4.62		+0.02 (SE 0.22)	0.92

SE = standard error; SWM = Semmes–Weinstein monofilaments; SWM categories = green, normal touch (1.65–2.83); blue, diminished light touch (3.22–3.61); purple, diminished protective sensation (3.84–4.31); red, loss of protective sensation (4.56–6.45); and red, deep pressure sensation only (6.65)

**Table 5:** Comparisons of breast sensation with and without nerve coaptation within the group without a previous implant

	Without nerve coaptation	SWM category	With nerve coaptation	SWM category	Absolute difference (= without − with nerve coaptation)	*p* value
T1 (*n* = without/with)						
Mean native (*n* = 71/87)	4.69		4.39		−0.30 (SE 0.17)	0.07
Mean flap (*n* = 71/87)	5.61		5.34		−0.27 (SE 0.10)	0.007*
Mean total (*n* = 71/87)	5.27		4.99		−0.28 (SE 0.11)	0.01*
T2 (*n* = without/with)						
Mean native (*n* = 71/87)	4.38		3.95		−0.43 (SE 0.15)	0.005*
Mean flap^a^ (*n* = 69/86)	5.18		4.72		−0.46 (SE 0.13)	<0.001*
Mean total (*n* = 71/87)	4.89		4.46		−0.43 (SE 0.11)	<0.001*
Tmax (*n* = without/with)						
Mean native (*n* = 71/87)	4.17		4.02		−0.15 (SE 0.16)	0.37
Mean flap^a^ (*n* = 69/86)	5.00		4.58		−0.42 (SE 0.13)	0.001*
Mean total (*n* = 71/87)	4.67		4.39		−0.28 (SE 0.11)	0.02*

SE = standard error; SWM = Semmes–Weinstein monofilaments; SWM categories = green, normal touch (1.65–2.83); blue, diminished light touch (3.22–3.61); purple, diminished protective sensation (3.84–4.31); red, loss of protective sensation (4.56–6.45); and red, deep pressure sensation only (6.65)

^*^ *p* < 0.05 indicating statistical significance

^a^ Two patients/three breasts missing postoperative flap values due to skin island removed in secondary procedure

## Discussion

In this cohort study, we compared sensation of the breast after DIEP flap reconstruction in patients with and patients without a prior IBBR to evaluate the influence of an implant or TE on the postoperative sensation of the replacing DIEP flap. The goal of this study was to further map out breast sensation after breast reconstruction. The outcomes are important to be able to better inform women preoperatively about what to expect of their postoperative breast sensation; questions which women ask on a regular basis at our outpatient clinic. We have analyzed breast sensation at different time points: T0, T1, T2, and Tmax, for the total breast skin, flap skin, and native skin. Our results show no significant influence of an implant on breast sensation comparing women with and without an implant prior to their DIEP flap reconstruction, even not after adjusting for the confounders of radiation therapy, BMI, diabetes, age, flap weight, length of follow-up, and performed nerve anastomosis. These results are further emphasized by the statistical insignificance of the SWM values seen in Table 3, as well as the visualization of the measurements in the scatterplots of Figs. 3, 4, and 5.

There is an increasing focus on postoperative breast sensation after reconstruction, especially since high levels of aesthetic result can be achieved with current techniques for autologous breast reconstruction. Many recent studies have focused on breast sensation after reconstruction. Several of these have shown the positive effect of improved sensation on quality of life and the importance of postoperative breast sensation is underlined by the recent development of the Sensation module of the BREAST-Q [10, 21, 22]. In addition to a DIEP flap reconstruction and as an alternative to implant reconstruction, Beugels et al. has demonstrated in a number of studies that recovery of breast sensation is positively influenced by performing a nerve coaptation accompanying the DIEP flap [14, 16]. This can especially be seen in Table 5, displaying the clinically and statistically significant effect of nerve coaptation in addition to the DIEP flap on postoperative breast sensation. We hypothesize the results found in Table 4 are not statistically significant due to the small number of included breasts, as the study was not powered for these outcomes. van Rooij et al. recently showed that swapping an implant reconstruction for a DIEP flap initially decreases postoperative sensation, but that sensation levels return to their preoperative value after approximately 1.5 years [17]. However, whether an implant does or does not influence postoperative sensation of the DIEP flap had not been studied before.

To analyze a hypothetical relationship between a previous implant and the sensation of the replacing DIEP flap, we have used a linear mixed-effects model analysis. Despite being statistically insignificant, the coefficients retrieved from the linear mixed-effects model were also not clinically relevant. None of the coefficients reached a value close to −1 or 1, with the largest deviation from zero being −0.15. Bijkerk et al. found that breast sensation decreases after mastectomy, but decreases even further after reconstructing the breast with an implant [11, 12]. They also found IBBR to be significantly associated with impaired sensation. Reflecting these results on our population, this could mean that the group with an implant prior to their DIEP flap could have worse breast sensation than the group without an implant prior to their DIEP flap. However, our results show no significant impact of an implant on the postoperative sensation of the DIEP flap, both at short and long-term follow-up. This indicates that the implant does not permanently damage the nerve endings in the skin and does not directly influence the sensory nerves or nerve regrowth of the replacing DIEP flap. When the implants are in place, they may act as a temporary physical barrier to block the sprouting nerves partially reinnervating the overlying skin [23]. However, after the implant has been removed and the DIEP flap is in place, the sensory innervation seems to start recovering from baseline again.

Our study has some limitations. First, we have not distinguished between TE and IBBR for our analysis. Nevertheless, we do not expect its effects on sensation to be different. Both have a similar outer shell and the decrease in sensation caused by an implant seems to be primarily caused by its barrier effect preventing nerve regrowth to the surface, based on our results and previous studies [12]. One might argue that the volume of the implant or inflation of the TE can cause extra nerve or skin damage, but van Rooij et al. have found no correlation between implant volume and breast sensation when the implant is in place [17]. Second, the data used in our study has been collected prospectively for another study but has been retrospectively analyzed for the current study. Hence, we have included as much patients as possible but have not performed a power analysis specifically for this study. The number of patients included in combination with a large number of confounders added to the multivariate logistic regression analysis may have influenced the statistical significance of our study. Last, the intrarater and interrater-reliability of the SWM has been debated [24]. To increase reliability of the measurements and decrease of bias, we have used the same two experienced researchers for performing the sensation measurements.

Future studies should further focus on the underlying physiology of sensation of the breast after autologous reconstruction and, especially, focus on discovering techniques to further improve the sensation of the breast. As stated earlier, improvement in sensation means improvement in breast-related quality of life, which makes it paramount in optimizing the outcomes of breast reconstruction. In our academic hospital, we are currently conducting a double-blinded, randomized controlled trial to compare the quality of life and objectively measured sensation of the reconstructed breast in women with and without nerve coaptation accompanying the DIEP flap.

## Conclusions

In conclusion, an implant preceding a DIEP flap reconstruction does not influence the postoperative sensation or recovery of sensation of the reconstructed breast, even after adjusting for the confounders radiation therapy, BMI, diabetes, age, flap weight, length of follow-up and performed nerve anastomosis. The results of this study can be used in patient counseling and providing women with information about what to expect regarding breast sensation after autologous breast reconstruction.

## Data Availability

The data that support the findings of this study are not openly available due to reasons of sensitivity and are available from the corresponding author upon reasonable request. Data are located in controlled access data storage at the Maastricht University Medical Center.
